# Subjective Sleep Timing and Social Jet Lag Relate to Neural Activation During Reward Feedback in Adolescence

**DOI:** 10.1111/jsr.70355

**Published:** 2026-06-01

**Authors:** Alyssa J. Parker, Jillian Lee Wiggins, Surabhi Bhutani

**Affiliations:** ^1^ Department of Psychology University of Maryland College Park Maryland USA; ^2^ Psychology Department San Diego State University San Diego California USA; ^3^ School of Exercise and Nutritional Sciences San Diego State University San Diego California USA

## Abstract

During adolescence, circadian phase delay yields later sleep timing and greater social jetlag (SJL), coinciding with heightened reward‐circuit reactivity as subcortical systems mature before prefrontal control. Evidence linking sleep timing to reward‐related neural activation in youth is limited. We examined whether sleep timing and SJL are associated with neural activation during reward processing in adolescents. In individuals with fMRI data, mid‐sleep on free days (MSFsc) and SJL were derived from the Munich Chronotype Questionnaire (MCTQ; *n* = 5678) and Fitbit data (*n* = 2245) in adolescents (10–13 years) from the ABCD dataset. Reward processing was assessed using the fMRI Monetary Incentive Delay task, dissociating anticipation (reward/loss vs. neutral) and feedback (hit vs. miss). Linear mixed‐effects models relate BOLD activation in a priori regions (amygdala, striatum, orbitofrontal cortex [OFC], insula, cingulate and frontal subregions) to sleep metrics. Later self‐reported MSFsc was associated with greater activation during reward receipt in the left amygdala and right lateral OFC (*β* = 0.05–0.06, *p*_fdr_ < 0.05). The OFC association persisted after adjustment for sleep duration, while the amygdala effect was attenuated. Higher self‐reported SJL also predicted increased left amygdala activation during reward receipt (*β* = 0.05, *p*_fdr_ < 0.05). Fitbit‐derived MSFsc and SJL were not associated with reward‐related activation after correction for multiple comparisons. Self‐reported later sleep timing and greater SJL were linked to heightened affective and regulatory neural responses during reward–feedback. The divergence between self‐reported and Fitbit‐derived findings suggests that subjective and objective sleep timing measures may capture distinct aspects of sleep timing with relevance for neural reward outcomes. Sleep timing, beyond duration, represents a modifiable target for improving adolescent circadian health.

## 1 Introduction

Adolescence is a period of rapid brain maturation when sufficient, well‐timed sleep is critical for cognitive, emotional and physical health (Logan et al. 2018). This developmental phase is marked by notable shifts in sleep timing, characterised by a preference for later bed and wake times (eveningness) (Logan et al. 2018). These shifts are driven by a developmental delay in melatonin secretion and heightened sensitivity to evening light, often extending bedtimes by 2–3 h relative to childhood (Gradisar et al. 2011; Pifer et al. 2024). Although this circadian delay is normative (Crowley et al. 2014; Roenneberg et al. 2004; Thorleifsdottir et al. 2002; Yang et al. 2005), its negative consequences are amplified by social constraints such as early school start times, creating a misalignment between adolescents' biological clocks and societal demands, termed social jetlag (SJL). Greater eveningness and SJL have been consistently associated with increased risks for obesity, cardiometabolic dysfunction, mood disorders and substance use (Borisenkov et al. 2022; Owens et al. 2010; Touitou 2013; Hayes et al. 2018; Jarrin et al. 2013; Morrissey et al. 2020). Identifying the pathways by which circadian misalignment affects adolescent health is therefore critical for developing targeted interventions and is a public health priority.

Adolescence also coincides with significant development in neural circuits involved in reward processing, including anticipation, receipt and loss. Reward sensitivity heightens as subcortical reward circuits mature earlier relative to prefrontal regulatory areas (Mills et al. 2014). This neurodevelopmental imbalance predisposes adolescents to risk‐taking and sensation‐seeking behaviours (Spear 2000). Previous neuroimaging studies in this life stage also link greater eveningness with reduced medial prefrontal cortex activation (Forbes et al. 2012) and increased ventral striatal responses during reward anticipation (Forbes et al. 2012; Hasler et al. 2013), signalling diminished regulatory control alongside enhanced reward drive (Gee et al. 2018; Murray et al. 2009; Venkatraman et al. 2011). Experimentally induced circadian misalignment in healthy adolescents similarly demonstrates reduced neural responsiveness to monetary rewards and impaired inhibitory control (Hasler et al. 2021). This underscores delayed sleep timing as a distinct factor influencing reward processing. However, literature in the highly sensitive stage of adolescence is limited and rarely examines real‐world sleep. Most evidence is derived from adults in laboratory settings, thus highlighting the need to extend these findings to adolescents in naturalistic sleep environments.

A critical gap also remains in understanding how reward processing relates to subjective versus objective indicators of sleep timing and SJL in adolescents. Self‐reported and actigraphy‐based sleep measures provide complementary perspectives on adolescent sleep patterns (Kiss et al. 2025). Subjective (i.e., self‐report) measures reflect perceived sleep behaviours shaped by social and psychological contexts, whereas objective (i.e., actigraphy) measures capture physiological sleep timing in naturalistic settings. Comparing these approaches is essential to clarify how perceived versus physiological sleep timing differentially relates to neural and behavioural outcomes. This dual‐method approach enables more precise identification of mechanisms through which circadian misalignment influences brain development and reward‐related processes during adolescence (Kiss et al. 2025).

To address these gaps, we leveraged data from the Adolescent Brain Cognitive Development (ABCD) Study, a large, diverse cohort of youths from the United States. The ABCD study uniquely allows for examining relationships between development, sleep and reward processing within a narrow age range, controlling for context‐related confounds. This dataset integrates comprehensive objective (actigraphy/Fitbit) and subjective (self‐report) sleep assessments collected in naturalistic environments, along with comprehensive demographic and developmental measures (age, puberty, socioeconomic status and sex). We used the Monetary Incentive Delay (MID) task to evaluate distinct anticipation (potential reward or loss) and feedback (actual reward receipt or loss outcomes) phases.

In the current study, the primary objective was to test whether habitual sleep timing (self‐report) and SJL are associated with neural activation during reward anticipation and feedback in adolescents using the subset with usable MID fMRI data. The second objective was to repeat the same fMRI models using Fitbit‐derived sleep timing/SJL in the smaller subset with valid Fitbit and usable fMRI data to evaluate whether observed sleep timing over a short recording window shows similar or different neural associations. We hypothesised that later sleep timing and greater SJL would be associated with altered neural activation across components of reward processing, reflecting heightened striatal responses and therefore increased reward sensitivity, along with reduced regulatory control in adolescents.

## 2 Methods and Materials

### 2.1 Participants

In the current study, the data were drawn from the ABCD study Year‐2 follow‐up (Release 5.1, DOI: https://doi.org/10.15154/z563‐zd24). The larger longitudinal data includes 11,868 children starting from ages 9 to 11 years recruited from 21 centres across the United States with a diverse range of geographic, socioeconomic, ethnic and health backgrounds. Because the primary research question concerns reward‐related neural activation, inferential analyses were conducted in the subset with usable MID fMRI data. Exclusion criteria were (Logan et al. 2018) reporting using an alarm to wake up on free days (*n* = 1433) and (Gradisar et al. 2011) unavailable or poor‐quality fMRI data (*n* = 5124). Of the 8123 participants with MID fMRI, 1379 were excluded for ABCD quality check criteria, yielding 6744 usable MID data points. The final analytic sample for primary models included 5678 for Munich Chronotype Questionnaire (MCTQ) and fMRI, and the final analytic sample for Fitbit models included 2245 (see Figure S1 for full flow and exclusion reasons). See Sections 2.2.1, 2.2.2 and 2.3.1 for details on analysis‐specific exclusion of participants. Informed consent from the primary caregiver and assent from the children were obtained. The study was approved by the institutional review boards of all local study sites.

### 2.2 Sleep Measures

#### 2.2.1 MCTQ

The MCTQ is a self‐report measure that assesses typical sleep and wake times on school days and free days (Roenneberg et al. 2003). Two variables of interest were calculated from this measure: mid‐sleep time (self‐reported MSFsc) and self‐reported SJL. Considering that sleep on free days is less constrained by social factors, self‐reported MSFsc was estimated as the midpoint of sleep on free days minus half of the difference between sleep duration on free days and the average sleep duration of the week to control for sleep debt (midpoint of sleep on school‐free days, sleep‐corrected and self‐reported MSFsc). Self‐reported SJL, a marker of circadian misalignment, was calculated as the absolute difference between the midsleep point on free days and the midsleep point on school days. Average nightly sleep duration was also extracted to serve as a covariate, allowing for the examination of sleep timing effects independently of sleep quantity. Variables were computed in R (Version 4.5; R Core Team 2025) using the MCTQ package (Vartanian et al. 2022).

Participants were excluded from MCTQ analyses if they were missing MCTQ data (*n* = 202), reported no free days (*n* = 86), were not regularly attending school (*n* = 144) or average sleep durations < 3 h or > 15 h (*n* = 43). Consistent with prior work using MSFsc to estimate preferred timing under minimal social constraint, we excluded participants who reported using an alarm to wake on free days (*n* = 1433). The resulting sample comprised 5678 participants.

#### 2.2.2 Fitbit‐Derived Sleep Measures

Objective sleep was measured in a subset of participants (*n* = 4968) using wrist‐worn Fitbit actigraphy data in the ABCD dataset. The objective sleep characteristics were collected over a 3‐week period, providing weekly summaries based on valid days (i.e., > 180 min of sleep). Fitbit‐derived mid‐sleep time (fitbit‐MSFsc), fitbit‐SJL and sleep duration were calculated using analogous methods to those applied to the MCTQ data. Participants were excluded from analyses if they had implausible average sleep durations (< 3 or > 15 h; *n* = 577), absence of free days data (*n* = 384) or had fewer than seven nights of valid sleep data (*n* = 1004). fMRI analyses are restricted to participants with both valid Fitbit timing estimates and usable MID fMRI data of 2245 participants.

Detailed descriptive comparisons between MCTQ‐ and Fitbit‐derived midsleep timing and SJL in the broader sleep samples (including correlations/mean differences) are provided in Table S1 and Supporting Information text to keep the main manuscript focused on the fMRI question.

### 2.3 fMRI Reward Task

Reward processing was measured through the MID task (for details, see Casey et al. 2018). The MID task consisted of two runs with 50 trials each. Each trial included three phases: (1) anticipation: cues indicated the potential to win money (reward condition), lose money (loss condition) or neither (neutral condition); (2) target: participants tried to respond quickly to hit a target; (3) feedback: participants were informed whether they hit or missed the target and thus received, lost or failed to receive a reward (depending on condition indicated by the cue). The task difficulty was adapted to performance throughout the task to produce an overall accuracy rate of approximately 60%.

Four contrasts were of interest: (1) reward anticipation (i.e., reward—neutral during anticipation), (2) loss anticipation (i.e., loss—neutral during anticipation), (3) reward receipt (i.e., hit—miss during reward trials during feedback) and (4) loss receipt (i.e., hit—miss during loss trials during feedback).

#### 2.3.1 Regions of Interest (ROIs)

A total of 13 ROIs in the striatum, limbic network, cingulate and frontal cortex were selected from the subcortical and Desikan–Killiany‐defined cortical pre‐tabulated data due to their previously observed associations with reward processing (Jauhar et al. 2021; Kahnt 2018). Specific ROIs included bilateral accumbens, amygdala, caudate, insula, lateral orbitofrontal cortex (OFC), medial OFC, parsopercularis, parsorbitalis, parstriangularis, rostral anterior cingulate cortex and superior frontal cortex. Outlier values, as defined by three standard deviations above or below the mean, were removed from each ROI prior to analyses. Participants were excluded from all analyses if they did not pass quality control checks implemented by the ABCD study. For details on ABCD data cleaning, preprocessing pipelines and quality control, see Hagler Jr. et al. (2019).

### 2.4 Data Analysis

Linear mixed‐effects models were used to examine associations between reward‐related activation and sleep timing measures using the lmer (Bates et al. 2015) and lmerTest (Kuznetsova et al. 2017) packages in R (R Core Team 2025). To predict sleep timing from neural activation during the MID task, models were run separately for each reward process (i.e., reward and loss anticipation and receipt) and each sleep timing measure (i.e., mid‐sleep time and SJL for self‐report and Fitbit); activation in all ROIs was included as predictors in all analyses. All analyses controlled for concurrent age, pubertal status, socioeconomic status (as measured by parental education) and sex. Analyses nested by family and scanner IDs to account for structural dependencies within the data. Analyses were run with and without controlling for sleep duration to evaluate its impact on results. *p* values were corrected using the false discovery rate (FDR) correction, as indicated by *p*_fdr_. Standardised coefficients (*β*) are reported as a measure of effect size.

Additional analyses examined the moderating role of youth sex assigned at birth on associations among reward‐related activation and sleep timing. See Table S2 for the results of these analyses.

## 3 Results

### 3.1 Descriptives

Table 1 presents a summary of the descriptive statistics for the dataset, showing a racially/ethnically and socioeconomically diverse, sex‐balanced sample with ages ranging from 10 to 13 years. The comparison of sleep measures collected using self‐reported and objective measures is described in Supporting Information.

**TABLE 1:** Sample characteristics.

	*N*	%
Sex assigned at birth (*N* = 5747)		
Female	2615	45.50%
Male	3132	54.50%
Race + ethnicity (*N* = 5747)		
Asian	106	1.84%
Black	657	11.43%
Hispanic or Latino/a	1076	18.72%
Indigenous/other	54	0.94%
White	3325	57.86%
Biracial or Multiracial	512	8.91%
Refused/missing/do not know	17	0.30%
Parental education (*N* = 5550)		
No 4‐year degree	1967	35.44%
4‐year degree or higher	3583	64.56%

	*M*	SD
Age (*N* = 5561)	11.95	0.65
Pubertal status (*N* = 5556)	2.11	0.70
MCTQ sleep onset time^a^ (*N* = 5678)	−1.50	1.31
MCTQ sleep–awake time^a^ (*N* = 5678)	7.00	1.45
MCTQ mid‐sleep time^a^ (*N* = 5678)	3.73	1.71
MCTQ social jetlag (*N* = 5678)	1.87	1.42
MCTQ average sleep duration (*N* = 5678)	8.84	1.25
Fitbit sleep onset time^a^ (*N* = 2245)	−1.51	2.66
Fitbit sleep awake time^a^ (*N* = 2245)	7.00	2.06
Fitbit mid‐sleep time^a^ (*N* = 2245)	2.26	3.16
Fitbit social jetlag (*N* = 2245)	1.98	2.43
Fitbit average sleep duration (*N* = 2245)	9.50	2.15

*Note:* Pubertal development was assessed using the Pubertal Development Scale (PDS).

Abbreviation: MCTQ, Munich Chronotype Questionnaire.

^a^ Times are reported in hours from 0 = *midnight*.

### 3.2 Neural Activations With Self‐Report‐Derived Mid‐Sleep Time (Self‐Reported‐MSFsc) and SJL (Self‐Reported‐SJL)

Self‐reported tendency for later sleep time (self‐reported MSFsc) was associated with greater activation when receiving compared to not receiving a reward in the left amygdala (*β* = 0.05, *t*(4797.03) = 2.89, *p* = 0.004, *p*_fdr_ = 0.024) and right lateral OFC (*β* = 0.06, *t*(4808.25) = 3.17, *p* = 0.002, *p*_fdr_ = 0.012; Figure 1). The association between self‐reported MSFsc and right lateral OFC activation survived after controlling for sleep duration (*β* = 0.05, *t*(4801.47) = 2.77, *p* = 0.006, *p*_fdr_ = 0.030); however, the association between self‐reported MSFsc and left amygdala activation when receiving versus not receiving a reward did not remain significant after controlling for sleep duration (*β* = 0.04, *t*(4788.96) = 2.38, *p* = 0.017, *p*_fdr_ = 0.078). We did not observe associations between self‐reported MSFsc and activation when anticipating potential loss or reward or when losing a reward.

**FIGURE 1:**
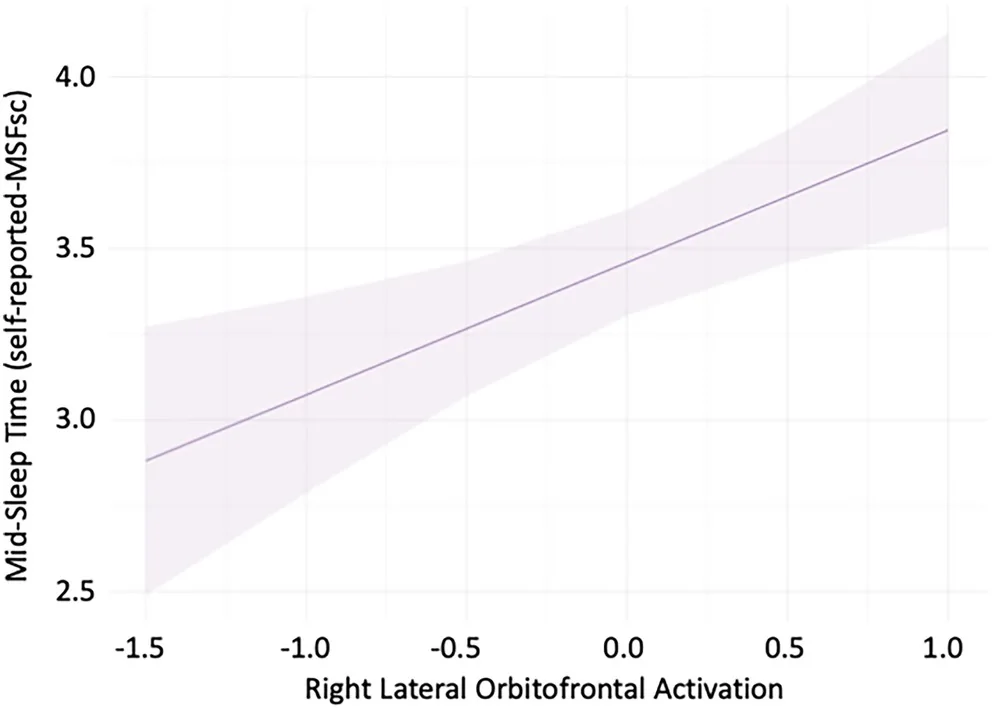
Association between right lateral OFC activation and mid‐sleep time presented as self‐reported MSFsc. *Note:* The same pattern was observed in the left amygdala. The figure with all individual data points is included as Figure S2.

Greater activation in the left amygdala when receiving compared to not receiving a reward was associated with greater self‐reported SJL (*β* = 0.05, *t*(4735.64) = 3.27, *p* = 0.001, *p*_fdr_ = 0.008). Associations between reward‐related left amygdala activation and SJL remained significant after controlling for sleep duration (*β* = 0.05, *t*(4744.03) = 3.17, *p* = 0.002, *p*_fdr_ = 0.010). There were no associations between self‐reported SJL and activation when anticipating loss or reward, nor when losing a reward.

### 3.3 Neural Activations With Objectively Measured Mid‐Sleep Time Fitbit‐MSFsc and SJL‐ACTG

In analysis restricted to the subset with usable MID fMRI data and valid Fitbit‐derived sleep timing, Fitbit‐derived MSFsc and SJL were not associated with reward‐related activation. See Table S1 for all results.

## 4 Discussion

Well‐timed sleep is foundational to multiple aspects of mental and physical health (Steardo et al. 2025), yet its association with transdiagnostic neural systems, such as reward circuitry that underlies such mental and physical health aspects (Ruge et al. 2024), remains underexplored. This gap is particularly salient during adolescence, a developmental “perfect storm” marked by delayed sleep timing, heightened reward neural sensitivity and the potential onset of mental disorders and physical conditions that may persist into adulthood (Dow‐Edwards et al. 2019). Here, we demonstrate the link between later sleep timing and greater reward neural sensitivity—above and beyond sleep duration—in a large, generalisable sample of adolescents undergoing critical developmental transitions. By integrating both self‐reported and objective assessments of sleep timing, our findings extend prior work and underscore the importance of circadian alignment for adolescent brain function.

Our key finding that habitual later sleep timing is associated with greater BOLD activity in affective (left amygdala) and regulatory (right lateral OFC) regions during monetary reward receipt builds on prior evidence linking circadian misalignment to heightened reward sensitivity in adolescence. Previous studies consistently demonstrate that later sleep patterns and higher SJL, hallmarks of circadian misalignment, correlate with heightened neural responsiveness to rewards in the ventral striatum, medial and lateral OFC and amygdala (Hasler et al. 2013, 2021; Zou et al. 2022). For example, Hasler and colleagues reported increased ventral striatal activation during reward receipt among adolescents with evening chronotype (preference for late sleep time), suggesting heightened reward sensitivity with delayed sleep timing emerging prominently in adolescence (Hasler et al. 2013, 2021; Goldstone et al. 2020). Similarly, other fMRI studies have shown that delayed sleep onset and circadian misalignment modulate mesolimbic and prefrontal function during reward processing (Holm et al. 2009), consistent with findings in reward (amygdala) and regulatory areas (lateral OFC) in the brain. Such enhanced reward responsivity among late sleepers carries clinical significance, as adolescence is a critical period marked by high neural plasticity in reward circuits, potentially increasing vulnerability to risk behaviours, impulsivity and mood disorders.

While most prior studies emphasise sleep duration, our findings highlight the distinctive and underexplored contribution of sleep timing and SJL, independent from sleep duration, in shaping neural reward responses. Shorter sleep duration is shown to be associated with altered activation in key reward‐related regions, notably the ventral striatum and medial prefrontal cortex and associated with increased risk‐taking behaviours and externalising symptoms in youth (Holm et al. 2009; Curtis et al. 2019). These studies employed fMRI paradigms such as the MID and gambling tasks (Curtis et al. 2019). Yet relatively few studies have specifically isolated delayed sleep timing from total sleep hours. The present findings clarify that circadian misalignment with delayed sleep timing influences reward circuitry above and beyond sleep duration. Distinguishing sleep timing from quantity provides a more nuanced understanding of timing, rather than quantity alone, as a distinctive pathway to altered neural reward processing in adolescence.

Contrary to some prior studies in adolescents and adults reporting circadian influences on neural activity during anticipation (Holm et al. 2009; Byrne et al. 2019), we did not observe significant associations between sleep timing or SJL and anticipatory reward activation in our adolescent sample. Byrne and colleagues highlighted that chronotype and circadian disruption can modulate mesolimbic and prefrontal activity across both anticipation and receipt phases of reward processing (Byrne et al. 2019). Similarly, reduced medial prefrontal cortex activation among habitual late sleepers during reward anticipation has been interpreted as evidence of diminished top–down regulatory control (Hasler et al. 2013). However, the literature demonstrates considerable variability in anticipation‐related findings, with directionality and region specificity modulated by pubertal developmental stage (Holm et al. 2009), age, measurement methods and reward phase (anticipation vs. outcome) (Holm et al. 2009; Hasler et al. 2022; Lunn et al. 2021), contributing to inconsistencies across studies. For example, Holm et al. observed that later sleep onset time predicted reduced caudate activation during both reward anticipation and outcome in early adolescents (Holm et al. 2009). Collectively, these inconsistencies underscore the need to clarify the developmental and contextual conditions under which delayed sleep timing alters neural mechanisms underlying reward anticipation. While both reward anticipation and receipt engage mesolimbic and prefrontal systems, our study suggests that sleep timing‐related circadian misalignment in early adolescence may more strongly relate to consummatory/outcome processing (feedback) than anticipatory responding.

A notable strength of our study is the inclusion of both subjective and objective sleep timing measures, which allowed us to test whether associations with reward‐related neural activation depend on how sleep timing and circadian misalignment are operationalised. Significant associations emerged specifically with MCTQ‐derived self‐reported midsleep and not with wrist‐worn device data. While null findings for an objectively recorded measure could seem unexpected, subjective and objective approaches likely capture overlapping but non‐identical aspects of sleep timing that differ in their relevance for reward‐related neural outcomes. The MCTQ was designed to characterise typical sleep timing separately for school days and free days, making it closely aligned with the conceptual definition of SJL as a socially structured form of circadian misalignment. In contrast, Fitbit‐derived estimates reflect observed sleep episodes over a limited recording window and may be more sensitive to short‐term schedule variability (e.g., atypical weeks), differences in proximity of the recording period to the fMRI session and device scoring algorithms for sleep onset/offset. Together, these factors could attenuate associations with neural outcomes even when sleep is measured objectively.

It is also important to note that the Fitbit sample was about half the size of the self‐reported MCTQ sample. Nevertheless, power analyses based on effect sizes observed with MCTQ suggested adequate power to detect comparable effects, making reduced sample size an unlikely sole explanation for the discrepancy. Overall, these findings support the value of multimethod sleep assessment in neuroimaging studies and suggest that habitual, socially constrained timing may be particularly relevant to reward–feedback neural responsivity in adolescence (Roenneberg et al. 2019).

While overall sleep–wake patterns were comparable across methods, with similar average sleep and wake times on school and free days across self‐report (MCTQ) and Fitbit. However, self‐reported mid‐sleep was on average 88 min later and self‐reported duration was 10 min longer than Fitbit‐derived estimates. In contrast, a recent ABCD analysis reported no MCTQ–Fitbit difference in mid‐sleep (Rohr et al. 2025). Our stricter exclusion criteria (e.g., removing youths using alarms on free days and those with missing/poor‐quality fMRI data) produced a smaller, more homogeneous sample, which may have improved the reliability of sleep‐timing estimates and contributed to these discrepancies. Methodological differences in sampling and filtering can yield both group‐level and individual‐level divergence between subjective and objective metrics. Consistent with prior adolescent and general‐population studies, subjective MCTQ assessments may produce a later mid‐sleep time and longer duration than objective measures of actigraphy or Fitbit, likely reflecting recall bias and aspirational schedule reporting (Santisteban et al. 2018). Ecological validity may also vary by chronotype, with greater recall variability in extreme morning and evening types (Lenneis et al. 2021). Despite these differences, SJL estimates converged across methods in our data, supporting SJL as a robust indicator of circadian misalignment in early adolescence, where social and biological pressures jointly shape sleep.

Strengths of the current investigation include the use of a large, nationally representative sample in a key age, multimodal sleep assessment and a validated fMRI reward paradigm. However, several limitations should be noted. Our cross‐sectional, correlational design does not allow us to infer directionality or make causal inferences. Future experimental, longitudinal and interventional studies are needed to examine causal pathways. Moreover, while leveraging a priori ROIs allows for focused insights, the ROI analyses limit the scope to specific brain areas, potentially missing important interactions and influences from other brain regions. Future research should include whole‐brain analyses to provide a more comprehensive examination of reward‐processing and sleep timing.

In summary, these findings underscore the critical clinical implications of circadian alignment in adolescent neural reward processing, distinct from sleep duration. Delayed sleep timing and SJL emerge as modifiable risk factors with significant implications for youth mental and behavioural health, thus highlighting the need for targeted prevention and policy strategies aimed at improving circadian health in adolescence. Adolescents experiencing greater SJL and later chronotypes are repeatedly shown to be at increased risk for mood disorders, substance use, impulsivity and weight dysregulation, partially mediated by altered reward processing (Logan et al. 2018; DePoy et al. 2024). Recent neuroimaging studies identify SJL as predictive of increased ventral striatal activity and reduced prefrontal engagement during reward tasks, which precedes negative mood trajectories and depressive symptoms in longitudinal data (Bates et al. 2015; Goldstone et al. 2020). These results suggest circadian misalignment is not only a correlate but also a potential intervention target for improving adolescent mental and behavioural health via public health strategies.

## Author Contributions

**Alyssa J. Parker:** conceptualization, methodology, software, formal analysis, visualization, writing – original draft, writing – review and editing, project administration, data curation, validation. **Jillian Lee Wiggins:** conceptualization, methodology, software, formal analysis, visulaization, writing – review and editing, supervision, project administration, data curation, validation. **Surabhi Bhutani:** conceptualization, methodology, formal analysis, writing – original draft, writing – review and editing, visualization, supervision, project administration, data curation, validation.

## Funding

This work was funded by R01MH122487 to Jillian Lee Wiggins. Data used in the preparation of this article were obtained from the Adolescent Brain Cognitive Development (ABCD) Study, held in the NIH Brain Development Cohorts Data Sharing Platform. This is a multisite, longitudinal study designed to recruit more than 10,000 children aged 9–10 and follow them over 10 years into early adulthood. The ABCD Study is supported by the National Institutes of Health and additional federal partners under award numbers U01DA041048, U01DA050989, U01DA051016, U01DA041022, U01DA051018, U01DA051037, U01DA050987, U01DA041174, U01DA041106, U01DA041117, U01DA041028, U01DA041134, U01DA050988, U01DA051039, U01DA041156, U01DA041025, U01DA041120, U01DA051038, U01DA041148, U01DA041093, U01DA041089, U24DA041123 and U24DA041147. A full list of supporters is available at https://abcdstudy.org/federal‐partners.html. A listing of participating sites and a complete listing of the study investigators can be found at https://abcdstudy.org/consortium_members/. ABCD consortium investigators designed and implemented the study and/or provided data, but did not necessarily participate in the analysis or writing of this report. This manuscript reflects the views of the authors and may not reflect the opinions or views of the NIH or ABCD consortium investigators.

## Ethics Statement

The study was approved by the institutional review boards of all local study sites for ABCD data collection.

## Consent

Informed consent from the primary caregiver and assent from the children were obtained.

## Conflicts of Interest

The authors declare no conflicts of interest.

## Supporting information


**Figure S1:** Flowchart of participant inclusion and exclusion criteria.
**Figure S2:** Association between right lateral OFC activation and mid‐sleep time presented as self‐reported‐MSFsc with individual data points.
**Table S1:** Full analysis results for associations between task‐related activation and midsleep time and SJL estimated using Fitbit and MCTQ.
**Table S2:** Moderation analysis results: Reward × sex assigned at birth.

## Data Availability

The data that support the findings of this study are available from the ABCD data repository. Restrictions apply to the availability of this data, which was used under licence for this study. Data are available from https://doi.org/10.15154/z563‐zd24.
