# Supplementary material for: Subjective Sleep Timing and Social Jet Lag Relate to Neural Activation During Reward Feedback in Adolescence
Source: J Sleep Res. 2026 Jun 1;35(5):e70355. doi: 10.1111/jsr.70355 (PMC13619011; doi:10.1111/jsr.70355)
Supplement: Supplementary file 1 [file JSR-35-e70355-s001.docx]

**Figure S1.** Flowchart of participant inclusion and exclusion criteria

**Figure S2.** Association between right lateral OFC activation and mid-sleep time presented as self-reported-MSFsc with individual data points.

**Comparison of Self-Reported and Objective Mid-Sleep Time and SJL Measures**

On average, participants self-reported falling asleep at 10:30PM (free days, 11:31PM; school days, 10:05PM) and waking at 7:00AM (free days, 7:50AM; school days, 6:39AM), with an adjusted mid-sleep time of 3:44AM. The average self-reported sleep duration was 8.84 hours, with duration on free days 46 min higher than school days (free days, 9.45 hours; school days, 8.59 hours). Participants had on average 1.87 hours of self-reported-SJL.

In comparison, Fitbit devices recorded participants falling asleep at 10:30PM (free days, 10:33PM; school days, 10:24PM) and waking at 6:53AM (free days, 7:11AM; school days, 6:48AM), with an adjusted mid-sleep time of 2:16AM. Fitbit-recorded sleep duration was 9.50 hours (free days, 9.81 hours; school days, 9.45 hours), with an average 1.98 hours of fitbit-SJL.

Overall, while the average sleep and wake time (free days and school days combined) were similar in self-reports and Fitbit, the adjusted mid-sleep time was 88 minutes later with self-report, compared to Fitbit. Similarly, the duration of sleep was 10 minutes longer for self-reported data, compared to Fitbit data. These discrepancies can be explained by differences in self-reported vs objective sleep and wake time on free days. The SJL metric was similar in both methods with an average of 115 minutes.

**Table S1.** Full analysis results for associations between task-related activation and midsleep time and SJL estimated using Fitbit and MCTQ.

|  | **MCTQ** | | | | | | | | | | | | | | **Fitbit** | | | | | | | | | | | | | |
| --- | --- | --- | --- | --- | --- | --- | --- | --- | --- | --- | --- | --- | --- | --- | --- | --- | --- | --- | --- | --- | --- | --- | --- | --- | --- | --- | --- | --- |
|  | **Original Analysis** | | | | | | | | | | **Controlling for Sleep Duration** | | | | **Original Analysis** | | | | | | | | | |  | **Controlling for Sleep Duration** | | |
|  | **Β** | | **t** | | | **df** | | **p** | | **p_fdr_** | **p** | | **p_fdr_** | **Β** | | | **t** | | | **df** | | **p** | | **p_fdr_** | | **p** | | **p_fdr_** |
| **MID SLEEP TIME** | | | |  | | |  | |  | |  |  | | |  |  | |  | | |  | |  | |  | |  | |
| **Reward Anticipation** | | | |  | | |  | |  | |  |  | | |  |  | |  | | |  | |  | |  | |  | |
| L. Accumbens | -0.03 | | -1.70 | | 4801.69 | | | 0.090 | | 0.382 | 0.104 | | 0.371 | 0.00 | | | -0.08 | | 1980.82 | | | 0.933 | | | 0.964 | 0.872 | | 0.973 |
| L. Amygdala | 0.02 | | 1.21 | | 4799.17 | | | 0.227 | | 0.540 | 0.486 | | 0.713 | 0.00 | | | -0.04 | | 1955.61 | | | 0.971 | | | 0.971 | 0.549 | | 0.973 |
| L. Caudate | 0.00 | | 0.11 | | 4752.45 | | | 0.910 | | 0.910 | 0.706 | | 0.807 | -0.04 | | | -0.61 | | 1958.59 | | | 0.544 | | | 0.943 | 0.713 | | 0.973 |
| L. Insula | -0.04 | | -1.38 | | 4804.12 | | | 0.168 | | 0.433 | 0.043 | | 0.231 | -0.01 | | | -0.31 | | 1973.64 | | | 0.756 | | | 0.943 | 0.528 | | 0.973 |
| L. Lateral Orbitofrontal | 0.01 | | 0.30 | | 4795.47 | | | 0.768 | | 0.880 | 0.433 | | 0.713 | 0.04 | | | 1.14 | | 1974.90 | | | 0.256 | | | 0.864 | 0.943 | | 0.973 |
| L. Medial Orbitofrontal | -0.01 | | -0.51 | | 4802.79 | | | 0.607 | | 0.758 | 0.916 | | 0.916 | -0.02 | | | -0.59 | | 1971.47 | | | 0.554 | | | 0.943 | 0.608 | | 0.973 |
| L. Parsopercularis | -0.03 | | -0.86 | | 4756.47 | | | 0.391 | | 0.638 | 0.321 | | 0.686 | -0.08 | | | -1.60 | | 1934.78 | | | 0.110 | | | 0.570 | 0.156 | | 0.809 |
| L. Parsorbitalis | 0.01 | | 0.38 | | 4763.11 | | | 0.706 | | 0.842 | 0.804 | | 0.880 | 0.01 | | | 0.20 | | 1976.85 | | | 0.845 | | | 0.964 | 0.998 | | 0.998 |
| L. Parstriangularis | 0.02 | | 0.70 | | 4809.93 | | | 0.484 | | 0.715 | 0.416 | | 0.713 | 0.02 | | | 0.47 | | 1962.40 | | | 0.640 | | | 0.943 | 0.378 | | 0.973 |
| L. Putamen | 0.00 | | -0.11 | | 4764.55 | | | 0.910 | | 0.910 | 0.621 | | 0.758 | 0.04 | | | 0.70 | | 1955.96 | | | 0.485 | | | 0.943 | 0.668 | | 0.973 |
| L. Rostral Anterior Cingulate | -0.04 | | -1.67 | | 4785.50 | | | 0.094 | | 0.382 | 0.139 | | 0.445 | 0.00 | | | 0.10 | | 1959.65 | | | 0.920 | | | 0.964 | 0.393 | | 0.973 |
| L. Rostral Middle Frontal | -0.06 | | -1.91 | | 4780.31 | | | 0.056 | | 0.382 | 0.102 | | 0.371 | 0.09 | | | 1.68 | | 1975.45 | | | 0.094 | | | 0.570 | 0.068 | | 0.434 |
| L. Superior Frontal | 0.06 | | 1.47 | | 4740.84 | | | 0.140 | | 0.433 | 0.197 | | 0.526 | 0.05 | | | 0.75 | | 1962.60 | | | 0.451 | | | 0.943 | 0.568 | | 0.973 |
| R. Accumbens | 0.03 | | 1.86 | | 4767.94 | | | 0.062 | | 0.382 | 0.064 | | 0.292 | 0.03 | | | 1.16 | | 1959.73 | | | 0.244 | | | 0.864 | 0.592 | | 0.973 |
| R. Amygdala | -0.01 | | -0.77 | | 4785.69 | | | 0.439 | | 0.681 | 0.566 | | 0.758 | -0.01 | | | -0.30 | | 1964.44 | | | 0.760 | | | 0.943 | 0.607 | | 0.973 |
| R. Caudate | -0.01 | | -0.21 | | 4748.91 | | | 0.833 | | 0.891 | 0.483 | | 0.713 | -0.01 | | | -0.09 | | 1955.64 | | | 0.925 | | | 0.964 | 0.872 | | 0.973 |
| R. Insula | 0.02 | | 0.59 | | 4780.07 | | | 0.553 | | 0.745 | 0.259 | | 0.636 | 0.05 | | | 1.00 | | 1968.18 | | | 0.318 | | | 0.864 | 0.476 | | 0.973 |
| R. Lateral Orbitofrontal | -0.01 | | -0.51 | | 4804.24 | | | 0.611 | | 0.758 | 0.572 | | 0.758 | -0.02 | | | -0.46 | | 1973.36 | | | 0.644 | | | 0.943 | 0.907 | | 0.973 |
| R. Medial Orbitofrontal | 0.02 | | 0.91 | | 4806.66 | | | 0.363 | | 0.638 | 0.639 | | 0.758 | -0.05 | | | -1.64 | | 1983.38 | | | 0.102 | | | 0.570 | 0.177 | | 0.809 |
| R. Parsopercularis | 0.02 | | 0.62 | | 4771.02 | | | 0.535 | | 0.745 | 0.613 | | 0.758 | 0.00 | | | -0.10 | | 1975.88 | | | 0.924 | | | 0.964 | 0.770 | | 0.973 |
| R. Parsorbitalis | 0.02 | | 0.87 | | 4772.11 | | | 0.382 | | 0.638 | 0.440 | | 0.713 | -0.03 | | | -1.00 | | 1958.30 | | | 0.320 | | | 0.864 | 0.234 | | 0.935 |
| R. Parstriangularis | -0.01 | | -0.26 | | 4754.85 | | | 0.795 | | 0.880 | 0.825 | | 0.880 | 0.02 | | | 0.37 | | 1927.91 | | | 0.712 | | | 0.943 | 0.815 | | 0.973 |
| R. Putamen | 0.03 | | 0.91 | | 4787.27 | | | 0.362 | | 0.638 | 0.490 | | 0.713 | -0.05 | | | -0.97 | | 1977.59 | | | 0.334 | | | 0.864 | 0.767 | | 0.973 |
| R. Rostral Anterior Cingulate | 0.03 | | 1.40 | | 4797.56 | | | 0.163 | | 0.433 | 0.186 | | 0.526 | -0.03 | | | -0.76 | | 1969.65 | | | 0.445 | | | 0.943 | 0.680 | | 0.973 |
| R. Rostral Middle Frontal | 0.04 | | 1.43 | | 4761.74 | | | 0.153 | | 0.433 | 0.315 | | 0.686 | -0.07 | | | -1.33 | | 1968.51 | | | 0.185 | | | 0.819 | 0.268 | | 0.951 |
| R. Superior Frontal | -0.04 | | -1.10 | | 4745.85 | | | 0.274 | | 0.565 | 0.427 | | 0.713 | 0.03 | | | 0.42 | | 1947.41 | | | 0.678 | | | 0.943 | 0.914 | | 0.973 |
| **Reward Feedback** |  |  | |  | | |  | |  | |  |  | | |  |  | |  | | |  | |  | |  | |  | |
| L. Accumbens | 0.02 | | 0.86 | | 4815.40 | | | 0.392 | | 0.578 | 0.413 | | 0.660 | -0.03 | | | -0.80 | | 1964.08 | | | 0.421 | | | 0.903 | 0.405 | | 0.967 |
| L. Amygdala | 0.05 | | 2.89 | | 4797.03 | | | 0.004 | | 0.024 | 0.017 | | 0.078 | -0.03 | | | -1.05 | | 1950.06 | | | 0.296 | | | 0.765 | 0.295 | | 0.943 |
| L. Caudate | -0.03 | | -0.96 | | 4765.50 | | | 0.337 | | 0.549 | 0.585 | | 0.711 | -0.03 | | | -0.46 | | 1929.37 | | | 0.647 | | | 0.903 | 0.974 | | 0.974 |
| L. Insula | -0.05 | | -1.91 | | 4763.09 | | | 0.056 | | 0.193 | 0.092 | | 0.267 | -0.09 | | | -1.87 | | 1943.69 | | | 0.061 | | | 0.476 | 0.040 | | 0.257 |
| L. Lateral Orbitofrontal | -0.03 | | -1.35 | | 4785.56 | | | 0.178 | | 0.393 | 0.153 | | 0.327 | 0.01 | | | 0.30 | | 1948.74 | | | 0.761 | | | 0.944 | 0.825 | | 0.974 |
| L. Medial Orbitofrontal | 0.03 | | 1.77 | | 4797.31 | | | 0.077 | | 0.218 | 0.104 | | 0.276 | -0.02 | | | -0.49 | | 1911.72 | | | 0.624 | | | 0.903 | 0.528 | | 0.967 |
| L. Parsopercularis | 0.04 | | 1.35 | | 4775.38 | | | 0.178 | | 0.393 | 0.213 | | 0.426 | 0.06 | | | 1.27 | | 1942.67 | | | 0.205 | | | 0.747 | 0.195 | | 0.779 |
| L. Parsorbitalis | 0.02 | | 0.91 | | 4782.68 | | | 0.361 | | 0.559 | 0.386 | | 0.651 | 0.04 | | | 1.24 | | 1945.30 | | | 0.217 | | | 0.747 | 0.342 | | 0.967 |
| L. Parstriangularis | 0.00 | | 0.09 | | 4742.84 | | | 0.926 | | 0.936 | 0.963 | | 0.963 | -0.06 | | | -1.38 | | 1933.12 | | | 0.167 | | | 0.740 | 0.435 | | 0.967 |
| L. Putamen | 0.07 | | 2.10 | | 4788.39 | | | 0.036 | | 0.160 | 0.079 | | 0.267 | 0.00 | | | 0.05 | | 1950.20 | | | 0.963 | | | 0.989 | 0.910 | | 0.974 |
| L. Rostral Anterior Cingulate | -0.03 | | -1.38 | | 4775.44 | | | 0.168 | | 0.393 | 0.136 | | 0.319 | -0.02 | | | -0.49 | | 1946.63 | | | 0.626 | | | 0.903 | 0.566 | | 0.967 |
| L. Rostral Middle Frontal | -0.03 | | -1.06 | | 4770.04 | | | 0.288 | | 0.499 | 0.516 | | 0.694 | -0.04 | | | -0.80 | | 1922.52 | | | 0.425 | | | 0.903 | 0.783 | | 0.974 |
| L. Superior Frontal | -0.02 | | -0.42 | | 4806.04 | | | 0.676 | | 0.737 | 0.526 | | 0.694 | 0.15 | | | 2.18 | | 1962.81 | | | 0.030 | | | 0.305 | 0.426 | | 0.967 |
| R. Accumbens | 0.00 | | -0.08 | | 4812.84 | | | 0.936 | | 0.936 | 0.881 | | 0.910 | -0.02 | | | -0.47 | | 1962.56 | | | 0.635 | | | 0.903 | 0.876 | | 0.974 |
| R. Amygdala | 0.02 | | 1.06 | | 4798.93 | | | 0.290 | | 0.499 | 0.139 | | 0.319 | 0.06 | | | 2.23 | | 1935.08 | | | 0.026 | | | 0.305 | 0.090 | | 0.434 |
| R. Caudate | 0.05 | | 1.31 | | 4748.78 | | | 0.190 | | 0.393 | 0.229 | | 0.431 | 0.01 | | | 0.13 | | 1902.46 | | | 0.893 | | | 0.989 | 0.935 | | 0.974 |
| R. Insula | -0.01 | | -0.43 | | 4779.51 | | | 0.667 | | 0.737 | 0.542 | | 0.694 | 0.05 | | | 1.04 | | 1951.87 | | | 0.296 | | | 0.765 | 0.478 | | 0.967 |
| R. Lateral Orbitofrontal | 0.06 | | 3.17 | | 4808.25 | | | 0.002 | | 0.012 | 0.006 | | 0.030 | -0.06 | | | -1.75 | | 1941.29 | | | 0.080 | | | 0.495 | 0.095 | | 0.434 |
| R. Medial Orbitofrontal | -0.04 | | -2.23 | | 4810.25 | | | 0.026 | | 0.133 | 0.085 | | 0.267 | 0.02 | | | 0.68 | | 1962.13 | | | 0.499 | | | 0.903 | 0.860 | | 0.974 |
| R. Parsopercularis | -0.01 | | -0.44 | | 4782.94 | | | 0.657 | | 0.737 | 0.456 | | 0.694 | 0.00 | | | 0.02 | | 1920.46 | | | 0.984 | | | 0.989 | 0.760 | | 0.974 |
| R. Parsorbitalis | 0.02 | | 0.82 | | 4755.39 | | | 0.414 | | 0.584 | 0.318 | | 0.565 | 0.00 | | | 0.08 | | 1939.26 | | | 0.938 | | | 0.989 | 0.909 | | 0.974 |
| R. Parstriangularis | -0.01 | | -0.40 | | 4774.12 | | | 0.689 | | 0.737 | 0.817 | | 0.872 | -0.02 | | | -0.43 | | 1917.40 | | | 0.670 | | | 0.903 | 0.574 | | 0.967 |
| R. Putamen | -0.06 | | -1.93 | | 4795.58 | | | 0.054 | | 0.193 | 0.052 | | 0.208 | -0.03 | | | -0.56 | | 1946.18 | | | 0.574 | | | 0.903 | 0.784 | | 0.974 |
| R. Rostral Anterior Cingulate | -0.01 | | -0.40 | | 4762.16 | | | 0.688 | | 0.737 | 0.531 | | 0.694 | 0.03 | | | 0.76 | | 1929.90 | | | 0.449 | | | 0.903 | 0.293 | | 0.943 |
| R. Rostral Middle Frontal | -0.01 | | -0.43 | | 4758.14 | | | 0.670 | | 0.737 | 0.711 | | 0.813 | 0.00 | | | 0.01 | | 1942.13 | | | 0.989 | | | 0.989 | 0.821 | | 0.974 |
| R. Superior Frontal | 0.02 | | 0.45 | | 4810.86 | | | 0.654 | | 0.737 | 0.600 | | 0.711 | -0.05 | | | -0.69 | | 1958.74 | | | 0.489 | | | 0.903 | 0.967 | | 0.974 |
| **SOCIAL JETLAG** |  | |  | |  | | |  | |  |  | |  |  | | |  | |  | | |  | | |  |  | |  |
| **Reward Anticipation** |  | |  | |  | | |  | |  |  | |  |  | | |  | |  | | |  | | |  |  | |  |
| L. Accumbens | -0.01 | | -0.31 | | 4732.58 | | | 0.755 | | 0.916 | 0.773 | | 0.932 | 0.00 | | | 0.04 | | 1970.68 | | | 0.971 | | | 0.971 | 0.852 | | 0.986 |
| L. Amygdala | 0.00 | | 0.27 | | 4746.60 | | | 0.790 | | 0.916 | 0.862 | | 0.932 | 0.01 | | | 0.30 | | 1904.01 | | | 0.764 | | | 0.971 | 0.724 | | 0.986 |
| L. Caudate | 0.00 | | 0.11 | | 4600.64 | | | 0.914 | | 0.916 | 0.883 | | 0.932 | 0.03 | | | 0.50 | | 1901.16 | | | 0.614 | | | 0.971 | 0.829 | | 0.986 |
| L. Insula | -0.04 | | -1.39 | | 4741.27 | | | 0.164 | | 0.916 | 0.134 | | 0.858 | 0.01 | | | 0.20 | | 1936.94 | | | 0.838 | | | 0.971 | 0.566 | | 0.986 |
| L. Lateral Orbitofrontal | -0.01 | | -0.33 | | 4726.65 | | | 0.745 | | 0.916 | 0.805 | | 0.932 | -0.04 | | | -1.36 | | 1939.94 | | | 0.173 | | | 0.922 | 0.855 | | 0.986 |
| L. Medial Orbitofrontal | 0.02 | | 1.09 | | 4757.84 | | | 0.276 | | 0.916 | 0.242 | | 0.932 | 0.01 | | | 0.18 | | 1944.13 | | | 0.857 | | | 0.971 | 0.946 | | 0.986 |
| L. Parsopercularis | -0.02 | | -0.73 | | 4613.63 | | | 0.467 | | 0.916 | 0.454 | | 0.932 | -0.03 | | | -0.53 | | 1839.36 | | | 0.597 | | | 0.971 | 0.305 | | 0.961 |
| L. Parsorbitalis | 0.01 | | 0.31 | | 4639.40 | | | 0.755 | | 0.916 | 0.765 | | 0.932 | -0.03 | | | -1.01 | | 1960.86 | | | 0.312 | | | 0.922 | 0.370 | | 0.986 |
| L. Parstriangularis | 0.01 | | 0.25 | | 4742.68 | | | 0.805 | | 0.916 | 0.790 | | 0.932 | -0.03 | | | -0.71 | | 1905.92 | | | 0.475 | | | 0.922 | 0.237 | | 0.844 |
| L. Putamen | 0.02 | | 0.54 | | 4642.55 | | | 0.589 | | 0.916 | 0.505 | | 0.932 | -0.04 | | | -0.79 | | 1902.36 | | | 0.427 | | | 0.922 | 0.588 | | 0.986 |
| L. Rostral Anterior Cingulate | 0.00 | | -0.15 | | 4694.97 | | | 0.878 | | 0.916 | 0.919 | | 0.932 | 0.03 | | | 0.73 | | 1930.44 | | | 0.463 | | | 0.922 | 0.997 | | 0.997 |
| L. Rostral Middle Frontal | -0.01 | | -0.45 | | 4691.76 | | | 0.650 | | 0.916 | 0.691 | | 0.932 | 0.05 | | | 1.07 | | 1945.85 | | | 0.283 | | | 0.922 | 0.200 | | 0.799 |
| L. Superior Frontal | -0.02 | | -0.43 | | 4562.51 | | | 0.665 | | 0.916 | 0.631 | | 0.932 | -0.01 | | | -0.14 | | 1908.62 | | | 0.887 | | | 0.971 | 0.903 | | 0.986 |
| R. Accumbens | 0.01 | | 0.60 | | 4654.68 | | | 0.550 | | 0.916 | 0.567 | | 0.932 | -0.01 | | | -0.44 | | 1900.92 | | | 0.662 | | | 0.971 | 0.686 | | 0.986 |
| R. Amygdala | 0.01 | | 0.72 | | 4697.14 | | | 0.474 | | 0.916 | 0.446 | | 0.932 | 0.00 | | | -0.08 | | 1919.35 | | | 0.939 | | | 0.971 | 0.923 | | 0.986 |
| R. Caudate | 0.01 | | 0.43 | | 4599.93 | | | 0.671 | | 0.916 | 0.734 | | 0.932 | -0.02 | | | -0.31 | | 1892.64 | | | 0.754 | | | 0.971 | 0.770 | | 0.986 |
| R. Insula | 0.00 | | 0.11 | | 4671.41 | | | 0.911 | | 0.916 | 0.854 | | 0.932 | 0.01 | | | 0.33 | | 1926.51 | | | 0.745 | | | 0.971 | 0.401 | | 0.986 |
| R. Lateral Orbitofrontal | 0.00 | | -0.12 | | 4738.94 | | | 0.908 | | 0.916 | 0.904 | | 0.932 | 0.02 | | | 0.77 | | 1927.61 | | | 0.444 | | | 0.922 | 0.922 | | 0.986 |
| R. Medial Orbitofrontal | -0.01 | | -0.33 | | 4770.18 | | | 0.745 | | 0.916 | 0.678 | | 0.932 | 0.00 | | | 0.09 | | 1970.70 | | | 0.931 | | | 0.971 | 0.679 | | 0.986 |
| R. Parsopercularis | 0.02 | | 0.64 | | 4663.00 | | | 0.525 | | 0.916 | 0.535 | | 0.932 | -0.01 | | | -0.22 | | 1944.60 | | | 0.824 | | | 0.971 | 0.477 | | 0.986 |
| R. Parsorbitalis | 0.00 | | -0.10 | | 4643.27 | | | 0.916 | | 0.916 | 0.887 | | 0.932 | 0.02 | | | 0.79 | | 1890.76 | | | 0.432 | | | 0.922 | 0.330 | | 0.961 |
| R. Parstriangularis | 0.03 | | 1.15 | | 4618.33 | | | 0.248 | | 0.916 | 0.248 | | 0.932 | 0.02 | | | 0.56 | | 1845.26 | | | 0.574 | | | 0.971 | 0.180 | | 0.799 |
| R. Putamen | -0.02 | | -0.66 | | 4705.46 | | | 0.512 | | 0.916 | 0.476 | | 0.932 | 0.04 | | | 0.71 | | 1953.49 | | | 0.475 | | | 0.922 | 0.955 | | 0.986 |
| R. Rostral Anterior Cingulate | 0.00 | | 0.11 | | 4728.55 | | | 0.916 | | 0.916 | 0.932 | | 0.932 | 0.00 | | | 0.11 | | 1933.04 | | | 0.912 | | | 0.971 | 0.653 | | 0.986 |
| R. Rostral Middle Frontal | 0.03 | | 1.09 | | 4622.64 | | | 0.274 | | 0.916 | 0.308 | | 0.932 | 0.04 | | | 0.71 | | 1933.46 | | | 0.476 | | | 0.922 | 0.689 | | 0.986 |
| R. Superior Frontal | -0.01 | | -0.33 | | 4579.45 | | | 0.741 | | 0.916 | 0.794 | | 0.932 | -0.07 | | | -1.03 | | 1883.31 | | | 0.305 | | | 0.922 | 0.462 | | 0.986 |
| **Reward Feedback** |  | |  | |  | | |  | |  |  | |  |  | | |  | |  | | |  | | |  |  | |  |
| L. Accumbens | 0.02 | | 0.87 | | 4787.71 | | | 0.387 | | 0.618 | 0.396 | | 0.654 | -0.01 | | | -0.42 | | 1956.54 | | | 0.678 | | | 0.930 | 0.603 | | 0.878 |
| L. Amygdala | 0.05 | | 3.27 | | 4736.63 | | | 0.001 | | 0.008 | 0.002 | | 0.010 | 0.04 | | | 1.43 | | 1938.27 | | | 0.152 | | | 0.509 | 0.137 | | 0.351 |
| L. Caudate | 0.02 | | 0.63 | | 4621.76 | | | 0.531 | | 0.715 | 0.490 | | 0.713 | 0.11 | | | 1.81 | | 1910.31 | | | 0.071 | | | 0.365 | 0.121 | | 0.351 |
| L. Insula | -0.03 | | -0.92 | | 4653.00 | | | 0.358 | | 0.618 | 0.387 | | 0.654 | -0.08 | | | -1.69 | | 1929.80 | | | 0.090 | | | 0.401 | 0.044 | | 0.175 |
| L. Lateral Orbitofrontal | -0.04 | | -2.20 | | 4708.12 | | | 0.028 | | 0.108 | 0.027 | | 0.095 | -0.01 | | | -0.21 | | 1935.64 | | | 0.837 | | | 0.930 | 0.900 | | 0.960 |
| L. Medial Orbitofrontal | 0.00 | | 0.24 | | 4740.59 | | | 0.812 | | 0.869 | 0.826 | | 0.886 | 0.04 | | | 1.38 | | 1889.06 | | | 0.168 | | | 0.509 | 0.700 | | 0.896 |
| L. Parsopercularis | 0.02 | | 0.85 | | 4660.78 | | | 0.393 | | 0.618 | 0.419 | | 0.654 | -0.01 | | | -0.29 | | 1928.33 | | | 0.773 | | | 0.930 | 0.898 | | 0.960 |
| L. Parsorbitalis | -0.01 | | -0.37 | | 4692.89 | | | 0.709 | | 0.843 | 0.700 | | 0.807 | -0.06 | | | -1.83 | | 1936.61 | | | 0.067 | | | 0.365 | 0.111 | | 0.351 |
| L. Parstriangularis | -0.02 | | -0.93 | | 4594.73 | | | 0.355 | | 0.618 | 0.352 | | 0.654 | 0.01 | | | 0.33 | | 1916.17 | | | 0.739 | | | 0.930 | 0.659 | | 0.896 |
| L. Putamen | 0.08 | | 2.54 | | 4704.55 | | | 0.011 | | 0.059 | 0.014 | | 0.060 | 0.07 | | | 1.34 | | 1943.47 | | | 0.181 | | | 0.509 | 0.154 | | 0.351 |
| L. Rostral Anterior Cingulate | -0.04 | | -1.68 | | 4675.81 | | | 0.092 | | 0.317 | 0.088 | | 0.277 | 0.01 | | | 0.32 | | 1934.31 | | | 0.749 | | | 0.930 | 0.728 | | 0.896 |
| L. Rostral Middle Frontal | -0.02 | | -0.63 | | 4670.57 | | | 0.529 | | 0.715 | 0.583 | | 0.750 | -0.01 | | | -0.20 | | 1910.10 | | | 0.840 | | | 0.930 | 0.367 | | 0.619 |
| L. Superior Frontal | -0.02 | | -0.59 | | 4739.47 | | | 0.557 | | 0.720 | 0.530 | | 0.737 | -0.02 | | | -0.28 | | 1957.25 | | | 0.782 | | | 0.930 | 0.165 | | 0.351 |
| R. Accumbens | -0.02 | | -0.98 | | 4779.94 | | | 0.325 | | 0.618 | 0.351 | | 0.654 | 0.02 | | | 0.70 | | 1956.96 | | | 0.483 | | | 0.903 | 0.951 | | 0.981 |
| R. Amygdala | 0.02 | | 1.60 | | 4745.71 | | | 0.109 | | 0.339 | 0.095 | | 0.277 | 0.00 | | | 0.06 | | 1918.50 | | | 0.955 | | | 0.993 | 0.368 | | 0.619 |
| R. Caudate | -0.02 | | -0.52 | | 4609.57 | | | 0.605 | | 0.750 | 0.591 | | 0.750 | -0.05 | | | -0.85 | | 1879.62 | | | 0.393 | | | 0.813 | 0.497 | | 0.795 |
| R. Insula | -0.03 | | -1.11 | | 4693.25 | | | 0.266 | | 0.618 | 0.251 | | 0.636 | 0.08 | | | 1.62 | | 1942.92 | | | 0.106 | | | 0.412 | 0.020 | | 0.106 |
| R. Lateral Orbitofrontal | 0.05 | | 2.53 | | 4759.12 | | | 0.011 | | 0.059 | 0.015 | | 0.060 | 0.02 | | | 0.64 | | 1927.10 | | | 0.524 | | | 0.903 | 0.717 | | 0.896 |
| R. Medial Orbitofrontal | 0.00 | | -0.20 | | 4768.61 | | | 0.841 | | 0.869 | 0.925 | | 0.925 | 0.00 | | | -0.02 | | 1956.55 | | | 0.982 | | | 0.993 | 0.33 | | 0.62 |
| R. Parsopercularis | 0.00 | | -0.06 | | 4693.69 | | | 0.954 | | 0.954 | 0.916 | | 0.925 | -0.02 | | | -0.39 | | 1901.47 | | | 0.700 | | | 0.930 | 0.885 | | 0.960 |
| R. Parsorbitalis | 0.02 | | 0.85 | | 4627.24 | | | 0.396 | | 0.618 | 0.388 | | 0.654 | 0.00 | | | -0.01 | | 1921.81 | | | 0.993 | | | 0.993 | 0.994 | | 0.994 |
| R. Parstriangularis | 0.02 | | 0.84 | | 4675.12 | | | 0.398 | | 0.618 | 0.371 | | 0.654 | -0.01 | | | -0.30 | | 1900.42 | | | 0.762 | | | 0.930 | 0.784 | | 0.929 |
| R. Putamen | -0.08 | | -2.46 | | 4732.94 | | | 0.014 | | 0.061 | 0.015 | | 0.060 | -0.06 | | | -1.00 | | 1938.30 | | | 0.317 | | | 0.756 | 0.131 | | 0.351 |
| R. Rostral Anterior Cingulate | -0.01 | | -0.34 | | 4653.72 | | | 0.734 | | 0.843 | 0.706 | | 0.807 | -0.03 | | | -0.88 | | 1910.14 | | | 0.378 | | | 0.813 | 0.243 | | 0.486 |
| R. Rostral Middle Frontal | 0.02 | | 0.78 | | 4631.79 | | | 0.435 | | 0.642 | 0.429 | | 0.654 | 0.04 | | | 0.68 | | 1927.85 | | | 0.496 | | | 0.903 | 0.566 | | 0.863 |
| R. Superior Frontal | 0.01 | | 0.20 | | 4765.72 | | | 0.841 | | 0.869 | 0.830 | | 0.886 | -0.04 | | | -0.57 | | 1948.96 | | | 0.569 | | | 0.929 | 0.161 | | 0.351 |

**Table S2.** Moderation Analysis Results: Reward X Sex Assigned At Birth

|  | **MCTQ** | | | | | | | | | | | | | | | **Fitbit** | | | | | | | | | | | | | | | | |
| --- | --- | --- | --- | --- | --- | --- | --- | --- | --- | --- | --- | --- | --- | --- | --- | --- | --- | --- | --- | --- | --- | --- | --- | --- | --- | --- | --- | --- | --- | --- | --- | --- |
|  | **Original Analysis** | | | | | | | | | | | **Controlling for Sleep Duration** | | | | **Original Analysis** | | | | | | | | | |  | | **Controlling for Sleep Duration** | | | | |
|  | **Β** | | **t** | | | **df** | | **p** | | **p_fdr_** | **p** | | | **p_fdr_** | **Β** | | **t** | | | | **df** | | **p** | | **p_fdr_** | | **p** | | | | **p_fdr_** | |
| **MID SLEEP TIME** | | | |  | | |  | |  | | |  |  | | |  | |  |  | | |  | |  | |  | | | |  | | |
| ***Reward Anticipation*** | | | |  | | |  | |  | | |  |  | | |  | |  |  | | |  | |  | |  | | | |  | | |
| L. Accumbens | 0.00 | | -0.03 | | 4777.19 | | | 0.972 | | 0.973 | 0.682 | | | 0.738 | 0.05 | | 1.71 | | | 1954.22 | | | 0.088 | | | 0.366 | | | 0.030 | | | 0.215 |
| L. Amygdala | -0.01 | | -0.30 | | 4780.50 | | | 0.764 | | 0.977 | 0.913 | | | 0.948 | -0.03 | | -0.90 | | | 1931.07 | | | 0.371 | | | 0.943 | | | 0.717 | | | 0.960 |
| L. Caudate | -0.02 | | -0.61 | | 4703.19 | | | 0.541 | | 0.752 | 0.231 | | | 0.727 | 0.05 | | 0.79 | | | 1904.80 | | | 0.428 | | | 0.687 | | | 0.602 | | | 0.837 |
| L. Insula | 0.07 | | 2.60 | | 4764.52 | | | 0.009 | | 0.117 | 0.019 | | | 0.237 | 0.00 | | -0.07 | | | 1923.28 | | | 0.947 | | | 0.947 | | | 0.726 | | | 0.861 |
| L. Lateral Orbitofrontal | -0.05 | | -2.28 | | 4761.82 | | | 0.023 | | 0.190 | 0.041 | | | 0.301 | -0.01 | | -0.34 | | | 1941.15 | | | 0.733 | | | 0.916 | | | 0.873 | | | 0.910 |
| L. Medial Orbitofrontal | 0.01 | | 0.62 | | 4777.28 | | | 0.535 | | 0.752 | 0.611 | | | 0.727 | 0.01 | | 0.42 | | | 1936.45 | | | 0.671 | | | 0.916 | | | 0.636 | | | 0.837 |
| L. Parsopercularis | 0.06 | | 2.09 | | 4722.60 | | | 0.037 | | 0.228 | 0.048 | | | 0.301 | -0.01 | | -0.20 | | | 1885.25 | | | 0.843 | | | 0.917 | | | 0.758 | | | 0.861 |
| L. Parsorbitalis | 0.00 | | -0.03 | | 4721.47 | | | 0.973 | | 0.973 | 0.718 | | | 0.738 | 0.01 | | 0.21 | | | 1934.59 | | | 0.830 | | | 0.917 | | | 0.742 | | | 0.861 |
| L. Parstriangularis | -0.04 | | -1.40 | | 4763.55 | | | 0.161 | | 0.721 | 0.410 | | | 0.727 | 0.06 | | 1.43 | | | 1930.56 | | | 0.152 | | | 0.381 | | | 0.055 | | | 0.229 |
| L. Putamen | 0.02 | | 0.52 | | 4712.30 | | | 0.605 | | 0.756 | 0.561 | | | 0.727 | -0.05 | | -0.82 | | | 1917.88 | | | 0.410 | | | 0.687 | | | 0.288 | | | 0.571 |
| L. Rostral Anterior Cingulate | 0.01 | | 0.28 | | 4773.31 | | | 0.781 | | 0.930 | 0.505 | | | 0.727 | -0.12 | | -2.96 | | | 1933.16 | | | 0.003 | | | 0.039 | | | 0.023 | | | 0.215 |
| L. Rostral Middle Frontal | -0.02 | | -0.75 | | 4739.06 | | | 0.451 | | 0.752 | 0.589 | | | 0.727 | -0.24 | | -4.57 | | | 1930.29 | | | 0.000 | | | 0.000 | | | 0.000 | | | 0.000 |
| L. Superior Frontal | -0.03 | | -0.73 | | 4714.29 | | | 0.468 | | 0.752 | 0.533 | | | 0.727 | 0.09 | | 1.30 | | | 1931.40 | | | 0.195 | | | 0.440 | | | 0.283 | | | 0.571 |
| R. Accumbens | -0.01 | | -0.53 | | 4756.36 | | | 0.599 | | 0.756 | 0.404 | | | 0.727 | 0.06 | | 2.07 | | | 1937.64 | | | 0.038 | | | 0.265 | | | 0.034 | | | 0.215 |
| R. Amygdala | 0.00 | | 0.08 | | 4776.63 | | | 0.933 | | 0.973 | 0.738 | | | 0.738 | 0.04 | | 1.47 | | | 1940.95 | | | 0.142 | | | 0.381 | | | 0.297 | | | 0.571 |
| R. Caudate | 0.04 | | 0.96 | | 4713.38 | | | 0.337 | | 0.752 | 0.122 | | | 0.610 | -0.11 | | -1.63 | | | 1904.05 | | | 0.104 | | | 0.370 | | | 0.142 | | | 0.444 |
| R. Insula | -0.02 | | -0.91 | | 4727.00 | | | 0.362 | | 0.752 | 0.463 | | | 0.727 | -0.01 | | -0.22 | | | 1932.54 | | | 0.825 | | | 0.917 | | | 0.481 | | | 0.837 |
| R. Lateral Orbitofrontal | 0.02 | | 0.82 | | 4774.98 | | | 0.415 | | 0.752 | 0.437 | | | 0.727 | 0.05 | | 1.52 | | | 1905.00 | | | 0.129 | | | 0.381 | | | 0.200 | | | 0.500 |
| R. Medial Orbitofrontal | 0.02 | | 0.84 | | 4780.65 | | | 0.404 | | 0.752 | 0.230 | | | 0.727 | -0.04 | | -1.25 | | | 1953.25 | | | 0.211 | | | 0.440 | | | 0.199 | | | 0.500 |
| R. Parsopercularis | -0.03 | | -1.04 | | 4742.39 | | | 0.296 | | 0.752 | 0.459 | | | 0.727 | 0.03 | | 0.73 | | | 1947.19 | | | 0.467 | | | 0.687 | | | 0.586 | | | 0.837 |
| R. Parsorbitalis | 0.02 | | 0.95 | | 4738.65 | | | 0.343 | | 0.752 | 0.409 | | | 0.727 | 0.03 | | 0.77 | | | 1886.04 | | | 0.441 | | | 0.687 | | | 0.590 | | | 0.837 |
| R. Parstriangularis | 0.00 | | -0.19 | | 4728.27 | | | 0.846 | | 0.961 | 0.665 | | | 0.738 | -0.04 | | -1.02 | | | 1906.76 | | | 0.306 | | | 0.588 | | | 0.607 | | | 0.837 |
| R. Putamen | -0.09 | | -2.61 | | 4760.90 | | | 0.009 | | 0.117 | 0.006 | | | 0.149 | 0.00 | | -0.08 | | | 1939.01 | | | 0.940 | | | 0.947 | | | 0.984 | | | 0.984 |
| R. Rostral Anterior Cingulate | 0.02 | | 0.66 | | 4770.81 | | | 0.510 | | 0.752 | 0.527 | | | 0.727 | 0.08 | | 1.99 | | | 1948.33 | | | 0.046 | | | 0.265 | | | 0.095 | | | 0.338 |
| R. Rostral Middle Frontal | -0.03 | | -0.99 | | 4705.60 | | | 0.324 | | 0.752 | 0.389 | | | 0.727 | 0.10 | | 1.94 | | | 1946.07 | | | 0.053 | | | 0.265 | | | 0.050 | | | 0.229 |
| R. Superior Frontal | 0.06 | | 1.36 | | 4679.73 | | | 0.173 | | 0.721 | 0.473 | | | 0.727 | 0.03 | | 0.37 | | | 1934.32 | | | 0.711 | | | 0.916 | | | 0.854 | | | 0.910 |
| ***Reward Feedback*** |  |  | |  | | |  | |  | | |  |  | | |  | |  |  | | |  | |  | |  | | | |  | | |
| L. Accumbens | -0.03 | | -1.20 | | 4794.67 | | | 0.231 | | 0.486 | 0.320 | | | 0.723 | 0.00 | | 0.05 | | | 1926.66 | | | 0.957 | | | 0.958 | | | 0.299 | | | 0.872 |
| L. Amygdala | 0.02 | | 1.28 | | 4783.83 | | | 0.201 | | 0.522 | 0.334 | | | 0.695 | -0.01 | | -0.40 | | | 1925.51 | | | 0.690 | | | 0.889 | | | 0.582 | | | 0.988 |
| L. Caudate | 0.09 | | 2.52 | | 4737.30 | | | 0.012 | | 0.109 | 0.030 | | | 0.365 | -0.02 | | -0.31 | | | 1889.14 | | | 0.758 | | | 0.958 | | | 0.570 | | | 0.891 |
| L. Insula | 0.02 | | 0.54 | | 4748.65 | | | 0.591 | | 0.703 | 0.461 | | | 0.723 | 0.05 | | 0.93 | | | 1916.29 | | | 0.350 | | | 0.795 | | | 0.386 | | | 0.872 |
| L. Lateral Orbitofrontal | 0.03 | | 1.46 | | 4754.31 | | | 0.144 | | 0.411 | 0.147 | | | 0.408 | -0.02 | | -0.58 | | | 1906.76 | | | 0.561 | | | 0.958 | | | 0.522 | | | 0.891 |
| L. Medial Orbitofrontal | -0.04 | | -1.83 | | 4774.31 | | | 0.067 | | 0.335 | 0.083 | | | 0.365 | 0.00 | | 0.08 | | | 1913.16 | | | 0.935 | | | 0.958 | | | 0.700 | | | 0.966 |
| L. Parsopercularis | 0.03 | | 0.86 | | 4769.31 | | | 0.391 | | 0.652 | 0.550 | | | 0.724 | 0.00 | | 0.05 | | | 1917.10 | | | 0.958 | | | 0.958 | | | 0.811 | | | 0.966 |
| L. Parsorbitalis | 0.01 | | 0.28 | | 4759.10 | | | 0.777 | | 0.821 | 0.870 | | | 0.884 | -0.04 | | -1.10 | | | 1928.82 | | | 0.273 | | | 0.744 | | | 0.217 | | | 0.793 |
| L. Parstriangularis | -0.03 | | -0.90 | | 4735.27 | | | 0.366 | | 0.652 | 0.520 | | | 0.723 | -0.03 | | -0.61 | | | 1904.67 | | | 0.543 | | | 0.958 | | | 0.453 | | | 0.872 |
| L. Putamen | -0.06 | | -1.59 | | 4756.28 | | | 0.112 | | 0.411 | 0.101 | | | 0.365 | -0.01 | | -0.13 | | | 1900.17 | | | 0.897 | | | 0.958 | | | 0.971 | | | 0.997 |
| L. Rostral Anterior Cingulate | -0.02 | | -0.90 | | 4755.79 | | | 0.367 | | 0.652 | 0.400 | | | 0.723 | 0.02 | | 0.41 | | | 1930.07 | | | 0.683 | | | 0.958 | | | 0.690 | | | 0.966 |
| L. Rostral Middle Frontal | -0.02 | | -0.62 | | 4772.60 | | | 0.537 | | 0.676 | 0.468 | | | 0.723 | -0.08 | | -1.36 | | | 1919.32 | | | 0.173 | | | 0.744 | | | 0.807 | | | 0.966 |
| L. Superior Frontal | 0.01 | | 0.27 | | 4760.71 | | | 0.788 | | 0.821 | 0.679 | | | 0.738 | 0.11 | | 1.53 | | | 1911.41 | | | 0.127 | | | 0.744 | | | 0.222 | | | 0.793 |
| R. Accumbens | 0.00 | | -0.20 | | 4795.45 | | | 0.843 | | 0.843 | 0.645 | | | 0.738 | 0.00 | | -0.12 | | | 1924.76 | | | 0.908 | | | 0.958 | | | 0.215 | | | 0.793 |
| R. Amygdala | 0.01 | | 0.66 | | 4778.14 | | | 0.510 | | 0.676 | 0.669 | | | 0.738 | 0.06 | | 2.20 | | | 1910.56 | | | 0.028 | | | 0.548 | | | 0.003 | | | 0.078 |
| R. Caudate | -0.09 | | -2.38 | | 4760.40 | | | 0.017 | | 0.109 | 0.032 | | | 0.365 | 0.05 | | 0.71 | | | 1903.71 | | | 0.477 | | | 0.958 | | | 0.414 | | | 0.872 |
| R. Insula | -0.01 | | -0.46 | | 4766.63 | | | 0.647 | | 0.735 | 0.514 | | | 0.723 | -0.10 | | -2.02 | | | 1922.68 | | | 0.044 | | | 0.548 | | | 0.040 | | | 0.473 |
| R. Lateral Orbitofrontal | -0.05 | | -2.47 | | 4780.08 | | | 0.014 | | 0.109 | 0.103 | | | 0.365 | -0.04 | | -1.04 | | | 1902.53 | | | 0.298 | | | 0.744 | | | 0.422 | | | 0.872 |
| R. Medial Orbitofrontal | 0.05 | | 2.51 | | 4770.32 | | | 0.012 | | 0.109 | 0.047 | | | 0.365 | -0.01 | | -0.36 | | | 1939.77 | | | 0.720 | | | 0.958 | | | 0.558 | | | 0.891 |
| R. Parsopercularis | -0.02 | | -0.65 | | 4755.82 | | | 0.513 | | 0.676 | 0.884 | | | 0.884 | 0.06 | | 1.17 | | | 1904.64 | | | 0.243 | | | 0.744 | | | 0.102 | | | 0.639 |
| R. Parsorbitalis | -0.03 | | -1.45 | | 4685.98 | | | 0.148 | | 0.411 | 0.100 | | | 0.365 | 0.04 | | 1.09 | | | 1905.14 | | | 0.276 | | | 0.744 | | | 0.908 | | | 0.987 |
| R. Parstriangularis | 0.04 | | 1.39 | | 4743.13 | | | 0.164 | | 0.411 | 0.117 | | | 0.365 | -0.01 | | -0.10 | | | 1880.76 | | | 0.918 | | | 0.958 | | | 0.881 | | | 0.987 |
| R. Putamen | 0.05 | | 1.19 | | 4765.51 | | | 0.233 | | 0.486 | 0.348 | | | 0.723 | 0.03 | | 0.50 | | | 1914.57 | | | 0.621 | | | 0.958 | | | 0.763 | | | 0.966 |
| R. Rostral Anterior Cingulate | 0.02 | | 0.61 | | 4749.33 | | | 0.541 | | 0.676 | 0.480 | | | 0.723 | 0.00 | | -0.06 | | | 1892.20 | | | 0.953 | | | 0.958 | | | 0.997 | | | 0.997 |
| R. Rostral Middle Frontal | 0.05 | | 1.40 | | 4765.40 | | | 0.163 | | 0.411 | 0.367 | | | 0.723 | 0.07 | | 1.21 | | | 1921.07 | | | 0.228 | | | 0.744 | | | 0.329 | | | 0.872 |
| R. Superior Frontal | -0.03 | | -0.66 | | 4791.02 | | | 0.510 | | 0.676 | 0.657 | | | 0.738 | -0.13 | | -1.73 | | | 1940.42 | | | 0.083 | | | 0.693 | | | 0.057 | | | 0.473 |
| **SOCIAL JETLAG** |  | |  | |  | | |  | |  |  | | |  |  | |  | | |  | | |  | | |  | | |  | | |  |
| ***Reward Anticipation*** | | |  | |  | | |  | |  |  | | |  |  | |  | | |  | | |  | | |  | | |  | | |  |
| L. Accumbens | 0.00 | | -0.01 | | 4717.24 | | | 0.991 | | 0.991 | 0.943 | | | 0.979 | 0.08 | | 2.57 | | | 1948.37 | | | 0.010 | | | 0.083 | | | 0.008 | | | 0.068 |
| L. Amygdala | 0.03 | | 2.00 | | 4725.76 | | | 0.045 | | 0.993 | 0.037 | | | 0.986 | -0.03 | | -1.14 | | | 1913.42 | | | 0.253 | | | 0.957 | | | 0.312 | | | 0.983 |
| L. Caudate | -0.02 | | -0.49 | | 4516.43 | | | 0.624 | | 0.942 | 0.553 | | | 0.960 | -0.01 | | -0.14 | | | 1876.21 | | | 0.891 | | | 0.891 | | | 0.835 | | | 0.882 |
| L. Insula | -0.01 | | -0.55 | | 4676.50 | | | 0.583 | | 0.942 | 0.542 | | | 0.960 | -0.06 | | -1.22 | | | 1909.48 | | | 0.223 | | | 0.410 | | | 0.201 | | | 0.420 |
| L. Lateral Orbitofrontal | -0.02 | | -1.03 | | 4681.48 | | | 0.305 | | 0.942 | 0.338 | | | 0.960 | -0.02 | | -0.68 | | | 1931.02 | | | 0.499 | | | 0.693 | | | 0.576 | | | 0.758 |
| L. Medial Orbitofrontal | -0.01 | | -0.42 | | 4735.96 | | | 0.673 | | 0.942 | 0.660 | | | 0.960 | 0.06 | | 1.85 | | | 1933.47 | | | 0.065 | | | 0.202 | | | 0.064 | | | 0.200 |
| L. Parsopercularis | 0.03 | | 1.09 | | 4553.84 | | | 0.275 | | 0.942 | 0.287 | | | 0.960 | -0.06 | | -1.17 | | | 1845.27 | | | 0.241 | | | 0.410 | | | 0.285 | | | 0.475 |
| L. Parsorbitalis | -0.03 | | -1.78 | | 4573.86 | | | 0.074 | | 0.942 | 0.066 | | | 0.960 | 0.01 | | 0.25 | | | 1921.64 | | | 0.806 | | | 0.839 | | | 0.896 | | | 0.896 |
| L. Parstriangularis | -0.01 | | -0.40 | | 4652.61 | | | 0.687 | | 0.942 | 0.762 | | | 0.960 | 0.11 | | 2.43 | | | 1917.64 | | | 0.015 | | | 0.083 | | | 0.012 | | | 0.077 |
| L. Putamen | 0.00 | | 0.01 | | 4552.75 | | | 0.991 | | 0.991 | 0.979 | | | 0.979 | 0.02 | | 0.40 | | | 1901.93 | | | 0.690 | | | 0.839 | | | 0.715 | | | 0.882 |
| L. Rostral Anterior Cingulate | -0.02 | | -0.74 | | 4704.21 | | | 0.460 | | 0.942 | 0.496 | | | 0.960 | -0.09 | | -2.12 | | | 1928.52 | | | 0.034 | | | 0.122 | | | 0.057 | | | 0.200 |
| L. Rostral Middle Frontal | -0.01 | | -0.43 | | 4622.48 | | | 0.666 | | 0.942 | 0.703 | | | 0.960 | -0.23 | | -4.46 | | | 1921.81 | | | 0.000 | | | 0.000 | | | 0.000 | | | 0.000 |
| L. Superior Frontal | 0.04 | | 1.04 | | 4527.08 | | | 0.298 | | 0.942 | 0.294 | | | 0.960 | 0.11 | | 1.61 | | | 1914.31 | | | 0.108 | | | 0.270 | | | 0.127 | | | 0.319 |
| R. Accumbens | 0.01 | | 0.31 | | 4652.27 | | | 0.753 | | 0.942 | 0.800 | | | 0.960 | 0.10 | | 3.26 | | | 1923.82 | | | 0.001 | | | 0.014 | | | 0.001 | | | 0.016 |
| R. Amygdala | 0.00 | | 0.20 | | 4716.65 | | | 0.843 | | 0.991 | 0.807 | | | 0.960 | 0.07 | | 2.38 | | | 1934.70 | | | 0.017 | | | 0.083 | | | 0.024 | | | 0.099 |
| R. Caudate | 0.05 | | 1.20 | | 4537.14 | | | 0.230 | | 0.942 | 0.195 | | | 0.960 | -0.06 | | -0.85 | | | 1879.89 | | | 0.396 | | | 0.619 | | | 0.429 | | | 0.670 |
| R. Insula | 0.01 | | 0.55 | | 4579.35 | | | 0.585 | | 0.942 | 0.548 | | | 0.960 | -0.02 | | -0.48 | | | 1920.54 | | | 0.634 | | | 0.834 | | | 0.555 | | | 0.758 |
| R. Lateral Orbitofrontal | 0.01 | | 0.44 | | 4694.35 | | | 0.662 | | 0.942 | 0.679 | | | 0.960 | 0.04 | | 1.16 | | | 1872.86 | | | 0.246 | | | 0.410 | | | 0.281 | | | 0.475 |
| R. Medial Orbitofrontal | 0.01 | | 0.74 | | 4747.24 | | | 0.462 | | 0.942 | 0.432 | | | 0.960 | -0.05 | | -1.65 | | | 1953.48 | | | 0.099 | | | 0.270 | | | 0.102 | | | 0.282 |
| R. Parsopercularis | 0.00 | | -0.03 | | 4630.60 | | | 0.976 | | 0.991 | 0.977 | | | 0.979 | 0.06 | | 1.32 | | | 1942.96 | | | 0.188 | | | 0.410 | | | 0.201 | | | 0.420 |
| R. Parsorbitalis | 0.02 | | 1.26 | | 4583.74 | | | 0.207 | | 0.942 | 0.213 | | | 0.960 | 0.01 | | 0.30 | | | 1836.08 | | | 0.763 | | | 0.839 | | | 0.823 | | | 0.882 |
| R. Parstriangularis | 0.00 | | -0.02 | | 4584.05 | | | 0.985 | | 0.991 | 0.947 | | | 0.979 | -0.01 | | -0.32 | | | 1873.24 | | | 0.746 | | | 0.839 | | | 0.847 | | | 0.882 |
| R. Putamen | -0.05 | | -1.66 | | 4672.57 | | | 0.098 | | 0.942 | 0.095 | | | 0.960 | -0.07 | | -1.24 | | | 1936.57 | | | 0.216 | | | 0.410 | | | 0.224 | | | 0.430 |
| R. Rostral Anterior Cingulate | 0.01 | | 0.60 | | 4695.74 | | | 0.546 | | 0.942 | 0.546 | | | 0.960 | 0.03 | | 0.78 | | | 1945.82 | | | 0.438 | | | 0.644 | | | 0.509 | | | 0.749 |
| R. Rostral Middle Frontal | -0.04 | | -1.21 | | 4521.22 | | | 0.228 | | 0.942 | 0.242 | | | 0.960 | 0.12 | | 2.33 | | | 1939.36 | | | 0.020 | | | 0.083 | | | 0.022 | | | 0.099 |
| R. Superior Frontal | -0.01 | | -0.34 | | 4456.71 | | | 0.731 | | 0.942 | 0.639 | | | 0.960 | 0.02 | | 0.25 | | | 1922.23 | | | 0.806 | | | 0.839 | | | 0.838 | | | 0.882 |
| ***Reward Feedback*** |  | |  | |  | | |  | |  |  | | |  |  | |  | | |  | | |  | | |  | | |  | | |  |
| L. Accumbens | -0.02 | | -0.99 | | 4778.41 | | | 0.324 | | 0.901 | 0.344 | | | 0.938 | -0.04 | | -1.08 | | | 1920.34 | | | 0.280 | | | 0.648 | | | 0.170 | | | 0.662 |
| L. Amygdala | -0.01 | | -0.83 | | 4743.14 | | | 0.406 | | 0.686 | 0.368 | | | 0.683 | -0.01 | | -0.36 | | | 1915.31 | | | 0.719 | | | 0.919 | | | 0.698 | | | 0.899 |
| L. Caudate | 0.01 | | 0.16 | | 4588.23 | | | 0.876 | | 0.936 | 0.920 | | | 0.972 | -0.06 | | -0.86 | | | 1857.86 | | | 0.390 | | | 0.648 | | | 0.356 | | | 0.665 |
| L. Insula | -0.01 | | -0.50 | | 4646.62 | | | 0.616 | | 0.923 | 0.635 | | | 0.938 | 0.08 | | 1.70 | | | 1898.51 | | | 0.088 | | | 0.602 | | | 0.094 | | | 0.588 |
| L. Lateral Orbitofrontal | 0.01 | | 0.70 | | 4671.10 | | | 0.484 | | 0.923 | 0.493 | | | 0.938 | -0.03 | | -0.95 | | | 1881.13 | | | 0.341 | | | 0.648 | | | 0.345 | | | 0.665 |
| L. Medial Orbitofrontal | -0.01 | | -0.78 | | 4721.27 | | | 0.437 | | 0.911 | 0.465 | | | 0.938 | 0.06 | | 1.66 | | | 1899.60 | | | 0.096 | | | 0.602 | | | 0.082 | | | 0.588 |
| L. Parsopercularis | 0.06 | | 1.85 | | 4688.05 | | | 0.064 | | 0.402 | 0.072 | | | 0.453 | 0.01 | | 0.20 | | | 1898.89 | | | 0.845 | | | 0.919 | | | 0.891 | | | 0.928 |
| L. Parsorbitalis | 0.01 | | 0.43 | | 4671.10 | | | 0.665 | | 0.923 | 0.684 | | | 0.950 | -0.04 | | -1.30 | | | 1917.18 | | | 0.195 | | | 0.648 | | | 0.185 | | | 0.662 |
| L. Parstriangularis | -0.02 | | -0.91 | | 4605.63 | | | 0.365 | | 0.911 | 0.391 | | | 0.938 | -0.04 | | -0.85 | | | 1876.17 | | | 0.395 | | | 0.648 | | | 0.382 | | | 0.665 |
| L. Putamen | 0.00 | | -0.13 | | 4669.38 | | | 0.900 | | 0.936 | 0.901 | | | 0.972 | 0.04 | | 0.63 | | | 1871.10 | | | 0.526 | | | 0.680 | | | 0.505 | | | 0.665 |
| L. Rostral Anterior Cingulate | -0.02 | | -0.80 | | 4650.50 | | | 0.423 | | 0.911 | 0.434 | | | 0.938 | 0.04 | | 0.82 | | | 1919.23 | | | 0.415 | | | 0.648 | | | 0.432 | | | 0.665 |
| L. Rostral Middle Frontal | 0.02 | | 0.53 | | 4706.63 | | | 0.594 | | 0.923 | 0.606 | | | 0.938 | -0.03 | | -0.53 | | | 1909.09 | | | 0.598 | | | 0.680 | | | 0.844 | | | 0.918 |
| L. Superior Frontal | -0.02 | | -0.59 | | 4668.65 | | | 0.557 | | 0.923 | 0.569 | | | 0.938 | 0.01 | | 0.12 | | | 1890.62 | | | 0.901 | | | 0.938 | | | 0.997 | | | 0.997 |
| R. Accumbens | 0.03 | | 1.35 | | 4772.15 | | | 0.176 | | 0.729 | 0.192 | | | 0.705 | 0.06 | | 1.69 | | | 1915.19 | | | 0.091 | | | 0.602 | | | 0.041 | | | 0.514 |
| R. Amygdala | 0.00 | | 0.11 | | 4734.99 | | | 0.911 | | 0.936 | 0.936 | | | 0.972 | 0.06 | | 2.04 | | | 1891.75 | | | 0.041 | | | 0.602 | | | 0.031 | | | 0.514 |
| R. Caudate | 0.00 | | -0.10 | | 4660.45 | | | 0.918 | | 0.936 | 0.955 | | | 0.972 | 0.05 | | 0.76 | | | 1882.30 | | | 0.445 | | | 0.655 | | | 0.431 | | | 0.665 |
| R. Insula | 0.03 | | 1.05 | | 4693.10 | | | 0.292 | | 0.901 | 0.306 | | | 0.938 | -0.08 | | -1.52 | | | 1912.41 | | | 0.128 | | | 0.639 | | | 0.143 | | | 0.662 |
| R. Lateral Orbitofrontal | -0.06 | | -2.82 | | 4729.25 | | | 0.005 | | 0.119 | 0.007 | | | 0.180 | -0.05 | | -1.33 | | | 1877.31 | | | 0.185 | | | 0.648 | | | 0.221 | | | 0.665 |
| R. Medial Orbitofrontal | 0.05 | | 2.50 | | 4717.05 | | | 0.013 | | 0.158 | 0.016 | | | 0.205 | -0.04 | | -1.03 | | | 1935.92 | | | 0.303 | | | 0.648 | | | 0.278 | | | 0.665 |
| R. Parsopercularis | -0.05 | | -1.61 | | 4665.54 | | | 0.107 | | 0.537 | 0.127 | | | 0.635 | 0.06 | | 1.03 | | | 1880.76 | | | 0.303 | | | 0.648 | | | 0.276 | | | 0.665 |
| R. Parsorbitalis | -0.04 | | -2.01 | | 4515.98 | | | 0.045 | | 0.371 | 0.041 | | | 0.341 | 0.00 | | 0.05 | | | 1886.46 | | | 0.963 | | | 0.963 | | | 0.811 | | | 0.918 |
| R. Parstriangularis | 0.04 | | 1.27 | | 4635.62 | | | 0.204 | | 0.729 | 0.197 | | | 0.705 | 0.03 | | 0.61 | | | 1846.63 | | | 0.540 | | | 0.680 | | | 0.481 | | | 0.665 |
| R. Putamen | 0.00 | | 0.08 | | 4695.72 | | | 0.936 | | 0.936 | 0.972 | | | 0.972 | -0.07 | | -0.96 | | | 1895.50 | | | 0.338 | | | 0.648 | | | 0.309 | | | 0.665 |
| R. Rostral Anterior Cingulate | 0.00 | | -0.10 | | 4654.44 | | | 0.921 | | 0.936 | 0.925 | | | 0.972 | -0.02 | | -0.60 | | | 1863.42 | | | 0.551 | | | 0.680 | | | 0.569 | | | 0.690 |
| R. Rostral Middle Frontal | 0.00 | | 0.12 | | 4680.77 | | | 0.903 | | 0.936 | 0.958 | | | 0.972 | 0.05 | | 0.83 | | | 1908.71 | | | 0.407 | | | 0.648 | | | 0.468 | | | 0.665 |
| R. Superior Frontal | 0.02 | | 0.43 | | 4757.76 | | | 0.664 | | 0.923 | 0.638 | | | 0.938 | -0.04 | | -0.54 | | | 1936.22 | | | 0.586 | | | 0.680 | | | 0.580 | | | 0.690 |
